# Supplementary material for: MetaRibo-Seq measures translation in microbiomes
Source: Nat Commun. 2020 Jun 29;11:3268. doi: 10.1038/s41467-020-17081-z (PMC7324362; doi:10.1038/s41467-020-17081-z)
Supplement: Supplementary file 10 — Supplementary Data 7 [file 41467_2020_17081_MOESM10_ESM.zip › File2/Confidence_High_Taxonomy/29818_out.krona.html]

Javascript must be enabled to view this page.

members
magnitude
magnitudeUnassigned
count
unassigned
taxon
rank

29818\_out

2

1
2
superkingdom

1
phylum
1239

1
class
186801

1
186802
order


SRS055017\_contig\_number\_contig-100\_243.205049
1
species
1897049


SRS011084\_contig\_number\_contig-100\_13652.120221
1
